# ApoB-specific CD4^+^ T cells in atherosclerosis: MHC-II antigen presentation, T cell plasticity, and immune tolerance

**DOI:** 10.3389/fimmu.2026.1903006

**Published:** 2026-09-10

**Authors:** Xiaobo Cao, Chongjing Mu, Yi Xu

**Affiliations:** 1 Electrocardiography and Electroencephalography Room, Taizhou Second People’s Hospital Affiliated to Yangzhou University, Taizhou, Jiangsu, China; 2 The Affiliated Suzhou Hospital of Nanjing Medical University, Suzhou, China; 3 Department of Cardiology, Taizhou Second People’s Hospital Affiliated to Yangzhou University, Taizhou, Jiangsu, China

**Keywords:** apolipoprotein B, atherosclerosis, CD4^+^ T cells, immune tolerance, MHC-II antigen presentation, peptide vaccination, regulatory T cells, tetramer

## Abstract

Despite intensive lipid-lowering therapy, substantial residual cardiovascular risk persists, and antigen-specific adaptive immunity may contribute to the inflammatory burden that remains after lipid levels are controlled. Apolipoprotein B (ApoB)-containing lipoproteins accumulate in the arterial wall and undergo local modification. During this process, ApoB-containing material may be taken up by antigen-presenting cells, processed into self-peptides, and presented through MHC-II molecules to CD4^+^ T cells. This review focuses on ApoB antigen processing and presentation, the functional heterogeneity of ApoB-reactive CD4^+^ T cells, and emerging antigen-specific therapeutic approaches. Evidence from selected mouse models and human HLA-restricted systems indicates that ApoB-reactive T cells can acquire FoxP3^+^ regulatory features. However, under hyperlipidemic conditions and during advanced disease, these regulatory features may overlap with, or be replaced by, T-bet^+^, memory-like, and pro-inflammatory phenotypes. Human studies using HLA-DR-p18 tetramers, activation-induced marker assays in peripheral blood mononuclear cells, and single-cell TCR analyses have begun to define disease-associated differences in ApoB-reactive T-cell states. Even so, current data do not yet prove longitudinal transitions within the same T-cell clones. Interpretation is further limited by HLA restriction, the rarity of ApoB-reactive cells, and relatively small study cohorts. In preclinical models, ApoB peptide vaccination and mucosal tolerance approaches suggest that antigen-specific immunity can be shifted toward regulation without broad immunosuppression. Taken together, ApoB-specific immunity appears to reflect a changing balance between tolerance and inflammation, influenced by antigen burden, HLA background, antigen-presenting cell status, and lesion stage.

## 1 Introduction

Even with intensive low-density lipoprotein cholesterol lowering, many patients with atherosclerotic cardiovascular disease continue to face considerable residual risk, in part because inflammatory activity within plaques may persist after lipid levels are reduced (1, 2). Atherosclerosis is now understood as a lipid-driven chronic inflammatory disorder that begins with arterial retention of apolipoprotein B (ApoB)-containing lipoproteins and progresses through sustained crosstalk among endothelial cells, macrophages, vascular smooth muscle cells, B cells, and T cells (3, 4). Among these immune populations, CD4^+^ T cells are particularly relevant because they integrate antigen recognition with co-stimulatory and co-inhibitory signals, shape macrophage and B-cell responses, and may either promote or limit vascular inflammation depending on context (5). CD8^+^ T cells, by contrast, recognize peptides presented by MHC-I and can mediate cytotoxic and inflammatory effects in the vascular wall. In mouse models, ApoB-100 peptide-specific CD8^+^ T cells show effector-memory and cytolytic features, although immunization with selected peptides has also been reported to reduce atherosclerosis (6). By contrast, CD4^+^ T cells recognize peptides presented by MHC-II and mainly coordinate helper, regulatory, and inflammatory programs through cytokines and interactions with antigen-presenting cells (7, 8). The present review therefore focuses on the MHC-II-restricted ApoB-specific CD4^+^ T-cell axis. This antigen-specific axis places adaptive autoimmunity alongside lipid-driven innate inflammation as a contributor to disease persistence.

Vascular autoimmunity is not limited to ApoB. T cells isolated from human plaques can recognize oxidized LDL (9), plaque-derived CD4^+^ T cells respond to human heat shock protein 60 (10), and β_2_-glycoprotein I-reactive T cells have been identified in advanced carotid plaques (11). These findings support a broader repertoire of vascular self-antigens. ApoB is nevertheless the best-characterized target at the MHC-II-restricted CD4^+^ T-cell level because immunodominant epitopes have been mapped, HLA binding has been measured, and peptide-MHC-II tetramers permit direct detection of reactive cells (8, 12). This review therefore uses ApoB-specific CD4^+^ T cells as a tractable model of antigen-specific vascular autoimmunity.

ApoB is an especially plausible self-antigen because it is abundant in atherogenic lipoproteins and becomes retained and modified within the intima (4, 13). Antigen-presenting cells can take up ApoB-containing material, process it, and present ApoB-derived peptides on MHC-II molecules. Defined ApoB epitope studies directly demonstrate ApoB-specific MHC-II recognition (8), whereas the MHC-II-deficient mouse study establishes a broader protective role for MHC-II antigen presentation without identifying ApoB as the responsible antigen (14). In the broader MHC-II pathway, internalized proteins are degraded within endolysosomal compartments before peptide fragments are loaded onto MHC-II for recognition by CD4^+^ T cells (15). In atherosclerosis, this antigen-specific pathway should not be viewed as a substitute for innate inflammation. Instead, it may help organize and maintain inflammatory responses to a persistent arterial self-antigen, while also giving these responses features of immunological memory.

The role of ApoB-reactive CD4^+^ T cells depends strongly on disease context. Some cells display regulatory programs that may restrain harmful inflammation, whereas others develop effector, memory, follicular-helper-like, or dysfunctional states linked to plaque progression (16). Hyperlipidemia, chronic antigen exposure, inflammatory antigen-presenting cells, and local plaque cytokines may weaken an initially tolerogenic response. By contrast, antigen-specific approaches could shift ApoB-directed immunity toward regulation without causing broad immunosuppression. This review therefore addresses three related questions: how ApoB peptides are presented by MHC-II, how ApoB-specific CD4^+^ T-cell states evolve during disease, and how this pathway might be monitored or therapeutically redirected.

## 2 MHC-II antigen presentation of ApoB in atherosclerosis

### 2.1 Lipoprotein retention, uptake, and peptide loading

Lipoprotein retention is generally considered the first step in this pathway. LDL and other ApoB-containing remnant lipoproteins bind to arterial proteoglycans and accumulate most readily in regions exposed to disturbed flow. Experimental disruption of ApoB–proteoglycan binding has been shown to markedly reduce early atherosclerotic lesion formation (4). Once retained, these particles can undergo oxidation, aggregation, glycation, and enzymatic remodeling (17). In experimental systems, proteolysis makes LDL more susceptible to hydrolysis by group V secretory phospholipase A_2_ and secretory sphingomyelinase, thereby promoting particle fusion and strengthening proteoglycan binding (18). Macrophages internalize modified LDL mainly through SR-A and CD36, which together account for most modified-LDL degradation in double-knockout studies (19). They can also take up native LDL through macropinocytosis (20). Dendritic cells likewise accumulate lipid, but under hypercholesterolemic conditions they may preserve their ability to process antigen, present peptides, and prime CD4^+^ T cells (21). B cells could also participate in this process, although direct *in vivo* evidence that specific B-cell subsets present defined ApoB peptides is still limited (22). In professional antigen-presenting cells, ApoB-derived peptides enter the MHC-II pathway; human epitope mapping and plaque immunopeptidomics directly demonstrate HLA-II-bound ApoB peptides recognized by CD4^+^ T cells (12, 23). *Apoe*^−/−^ mice unable to present antigen on MHC-II develop more severe atherosclerosis and have fewer regulatory T cells despite lower systemic Th1/Th2 cytokine and immunoglobulin levels (14). This experiment establishes a protective role for MHC-II antigen presentation in atherosclerosis generally, but it does not identify ApoB as the antigen responsible for that effect.

Antigen presentation is therefore not a passive technical step. The identity and activation state of the antigen-presenting cell help determine whether ApoB recognition induces tolerance, inflammation, or ineffective stimulation. CD11c^+^ dendritic cells isolated from hypercholesterolemic mice retain antigen-processing and CD4^+^ T-cell-priming efficacy (21). Live imaging of explanted aortas further showed cognate interactions between arterial CD11c^+^ antigen-presenting cells and CD4^+^ T cells, followed by T-cell proliferation and IFN-γ/TNF-α production (24). These data establish functional local antigen presentation in the arterial wall, although they do not prove that every interacting clone is ApoB-specific. How lipid loading and metabolic stress modify ApoB-specific restimulation remains unresolved.

B cells add another layer of regulation, although the evidence for direct ApoB presentation remains limited. Antibodies and B-cell receptors can recognize ApoB-related and oxidized-lipid epitopes, and receptor-mediated uptake could, in principle, permit subsequent MHC-II presentation (22). In dyslipidemic mouse models, autoimmune Tfh responses promote B-cell activation and autoantibody production (25), but this study did not establish an ApoB-specific B-cell presentation pathway or anti-lipoprotein antibody response. Whether distinct B-cell subsets preferentially support protective or pathogenic ApoB-specific CD4^+^ T-cell programs therefore remains unresolved.

### 2.2 Sites, detection, and context-dependence of presentation

Antigen presentation can occur in artery-draining lymph nodes and within diseased arteries. DC-dependent CD4^+^ T-cell priming remains intact in secondary lymphoid tissues under hypercholesterolemic conditions (21), whereas mature lesions and adventitial artery tertiary lymphoid organs provide sites for local immune interactions (26, 27). Mature plaques contain macrophages, dendritic cells, T cells, B cells, and necrotic material that can sustain repeated antigen recognition (26, 27). Repeated local presentation is therefore hypothesized to sustain T-cell receptor signaling, clonal expansion, and phenotypic remodeling, but direct longitudinal evidence for ApoB-specific clones remains limited (27).

Peptide-MHC-II tetramers now permit direct identification of ApoB-specific CD4^+^ T cells in mice and humans. Kimura et al. identified the ApoB peptide p18, whose sequence is shared between human and mouse ApoB and which binds mouse I-A^b^ and a human HLA-DR molecule containing HLA-DRB1*07:01. Corresponding I-A^b^-p18 and HLA-DRB1*07:01-restricted p18 tetramers detected p18-specific CD4^+^ T cells in mice and human peripheral blood (8). Single-cell RNA sequencing combined with paired TCR reconstruction can then connect antigen specificity to transcriptional state, clonality, and tissue localization (28, 29). Together, these approaches show that ApoB-specific CD4^+^ T cells are heterogeneous and can occupy regulatory, memory-like, effector-like, and mixed states (8, 28, 29). Low precursor frequencies and HLA diversity nevertheless remain major technical limitations.

Human ApoB is a large protein containing multiple immunodominant and subdominant HLA-II epitopes, and HLA alleles differ in the peptide segments they present (12). Direct immunopeptidomic analysis of human carotid plaques recently identified HLA-DR-bound peptides and demonstrated ex vivo CD4^+^ T-cell responses to selected ApoB-100 peptides in 22–39% of patients; response magnitude correlated with plaque vulnerability (23). Oxidation, other post-translational modifications, and proteolysis may further alter peptide generation, abundance, and context. ApoB-specific immunity therefore comprises a family of related responses shaped by HLA genotype, antigen load, lesion stage, and antigen-presenting cell state rather than recognition of a single universal epitope.

**Figure 1:**
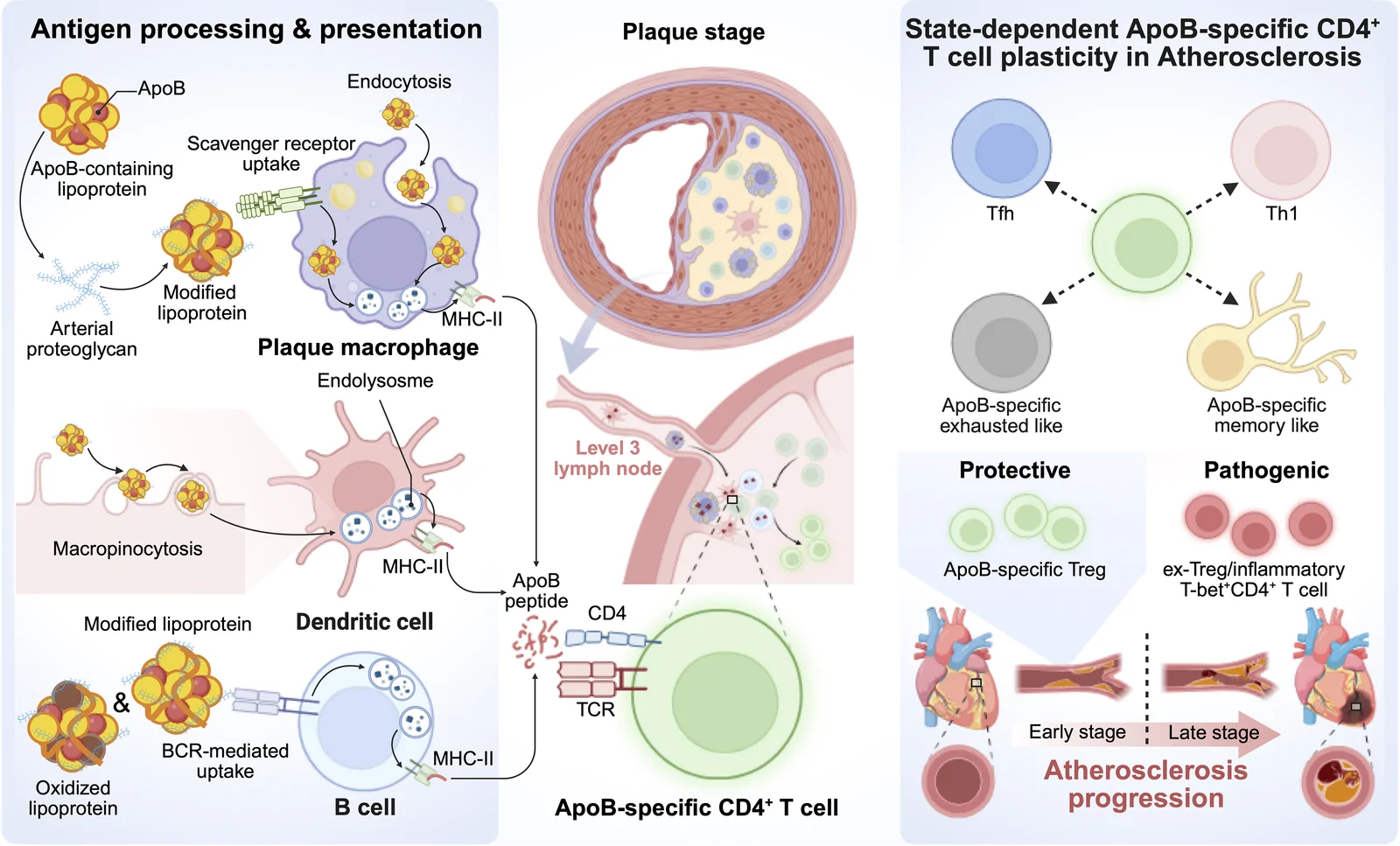
ApoB antigen presentation and CD4^+^ T cell plasticity in atherosclerosis.

Treatment may also alter the intensity and context of presentation. More intensive LDL lowering reduces the influx of ApoB-containing particles into the arterial wall, but residual lipoproteins, necrotic material, and inflammatory antigen-presenting cells may persist in established plaques. Anti-inflammatory therapy may reduce co-stimulation without directly removing antigen. ApoB-specific immunity is therefore jointly determined by antigen abundance, peptide processing, and the context in which peptide–MHC-II complexes are recognized (**Figure 1**).

## 3 ApoB-specific CD4^+^ T cell plasticity and plaque immunity

### 3.1 From tolerance to inflammation: phenotypic plasticity

The defining feature of ApoB-reactive CD4^+^ T cells is their plasticity. Among HLA-DRB1*07:01-positive individuals studied with an HLA-DR-p18 tetramer, p18-specific CD4^+^ T cells from those without cardiovascular disease exhibited prominent FoxP3^+^ regulatory features. This observation should not be generalized to every ApoB epitope or HLA background (8, 29). A 2025 activation-induced marker study of patients with angiographically verified coronary artery disease extended this model: ApoB-reactive cells were clonally expanded and enriched for tissue-homing effector Treg signatures in mild disease, whereas cells from participants with more severe disease showed weaker Treg programs and stronger glycolytic and interferon-response signatures (30). By analogy with broader Treg biology, regulatory ApoB-reactive cells may restrain macrophage activation and pro-inflammatory polarization, reduce cytokine production, and maintain self-tolerance. Direct ApoB-specific macrophage-interaction studies remain limited (31).

With disease progression, however, the same antigen-specific axis can become pathogenic. Hypercholesterolemia increases antigen burden and promotes inflammatory antigen-presenting cell states that expose T cells to IL-12, IL-6, IL-1 family cytokines, and TNF (32). In mouse studies, ApoB-responsive cells can acquire T-bet and aortic-homing chemokine receptors. CCR5^+^T-bet^+^FoxP3^+^ effector CD4^+^ T cells are associated with loss of the protective regulatory program and can drive atherosclerosis (16, 33). Evidence from non-antigen-specific T-cell models also implicates epigenetic regulation. CD4^+^ T-cell-specific deletion of *Ezh2* reduced atherosclerosis and shifted immunity toward Th2 and iNKT2 programs with anti-inflammatory macrophage polarization (34). This finding supports a general mechanism of T-cell plasticity but does not establish an ApoB-specific EZH2 pathway. Separately, interferon-γ-rich Th1 responses can increase MHC-II expression, activate inflammatory genes in plaque cells, suppress smooth-muscle-cell collagen synthesis, and potentially weaken plaque stability. These effects should not be attributed exclusively to ApoB-specific cells (35).

Effector states cannot be reduced to a single Th1 category. ApoB-specific cells can also exhibit Tfh-like, memory-differentiated, exhaustion-marker-positive, or transcriptionally mixed states. Tfh-like cells may regulate B-cell activation and autoantibody responses under dyslipidemic conditions (25), although direct evidence for ApoB-specific Tfh function in human plaques remains limited. A 2024 analysis of an expanded ApoB-reactive TCR dataset identified expanded clones and conserved CDR3 motifs that mapped preferentially to memory CD4^+^ T-cell repertoires and were shared across multiple donors, providing a potential route for tracking these cells beyond a single HLA-tetramer system (36). A memory phenotype, however, does not by itself establish recall function. Similarly, exhaustion markers may accompany persistent antigen exposure and TCR stimulation, but marker expression alone does not establish functional exhaustion (37). Mouse plaque single-cell RNA/TCR profiling has shown clonal expansion, exhaustion-associated programs, and local breakdown of tolerance checkpoints (38).

### 3.2 Reading plasticity: context, single-cell tools, and timing

A higher frequency of ApoB-specific CD4^+^ T cells should not be interpreted automatically as stronger pathogenic immunity. These cells may include protective Tregs, inflammatory Th1 cells, memory-like populations, or cells with impaired function. Their anatomical location is also important. Regulatory cells in draining lymph nodes are unlikely to have the same meaning as antigen-experienced cells within metabolically stressed plaques. In paired analyses of human carotid plaques and blood, clonal expansion was enriched among plaque-infiltrating effector CD4^+^ T cells, together with signs of recent TCR engagement, supporting a local antigen-driven response within lesions (39). Even so, spatially resolved functional validation is still needed (26, 27).

The plaque microenvironment contains many signals capable of reshaping immune-cell identity. Cholesterol crystals offer one well-defined example: they activate the NLRP3 inflammasome, and deletion of inflammasome components reduces atherogenesis in mice (40). Other plaque-associated cues, including hypoxia, oxidative stress, necrotic debris, and inflammatory lipids, can also modify macrophage and dendritic-cell states (32, 41). However, the direct contribution of each signal to ApoB-specific T-cell fate remains incompletely defined. Macrophages and dendritic cells that present ApoB peptides may also shape T-cell responses through co-stimulatory and co-inhibitory pathways. Mouse studies support a regulatory role for the PD-1/PD-L axis in atherosclerosis (42), whereas the effects of CD80/CD86, ICOS ligands, and related pathways on ApoB-specific T-cell differentiation are still mostly inferred from studies that did not directly track ApoB specificity (43).

Single-cell approaches have made it possible to connect antigen specificity with transcriptional state. Tetramer enrichment combined with paired TCR sequencing can reveal clonal expansion, shared receptor features, and disease-associated shifts in cell identity (29, 44). In the study by Saigusa et al., which included eight women, p18 tetramer-positive cells followed a regulatory-to-memory continuum in participants without subclinical disease, but shifted toward a more memory-like state in affected participants while retaining Treg-associated TCR features (29). Although these findings are informative, their interpretation is limited by HLA restriction, small cohort size, and the rarity of tetramer-positive cells. A practical working framework is therefore to group ApoB-specific CD4^+^ T cells into tolerogenic, inflammatory effector, helper-humoral, memory-like, exhaustion-like/dysfunctional, and transitional states (**Table 1**). These categories should not be regarded as fixed lineages, but as experimentally testable states that link antigen presentation, plaque biology, and candidate tolerance-based interventions (29, 30).

**Table 1:** Proposed states of ApoB-specific CD4^+^ T cells in atherosclerosis.

State	Key markers/features	Likely function	Evidence level	Caveats
Treg-like/tolerogenic	FoxP3^+^, IL-10, regulatory programs	Restrains macrophage activation and vascular inflammation	Mouse models; defined human p18-tetramer and AIM data	HLA- and epitope-limited; plaque stability remains uncertain
Th1-like inflammatory	T-bet^+^, IFN-γ, CCR5/CXCR6-related programs	Amplifies plaque inflammation and MHC-II expression	Mechanistic and mouse evidence; indirect human support	Not every Th1-like cell is ApoB-specific
Tfh-like helper-humoral	CXCR5^+^, B cell-help programs, antibody-related signatures	May shape B-cell activation and autoantibody responses under dyslipidemic conditions	Supported in dyslipidemic mouse models	Human plaque relevance remains unresolved
Memory-like	Antigen-experienced state; TCR persistence or clonal features	Could support recall responses	Human AIM/TCR and tetramer data	Phenotype does not prove recall function
Exhaustion-like/dysfunctional	Co-inhibitory or chronic-stimulation-associated signatures	May reflect persistent antigen exposure and reduced effector capacity	Mouse plaque data; limited ApoB-specific functional validation	Markers do not prove functional exhaustion
Transitional/hybrid	Mixed regulatory, effector, memory or Tfh-associated features	Captures movement between tolerance and inflammation	Cross-sectional single-cell data; no longitudinal fate validation	Analytical category, not a fixed lineage

The categories summarize currently proposed states rather than fixed lineages; evidence strength differs across mouse models, defined HLA/tetramer systems and human plaque studies.

This plasticity also makes timing critical. Early ApoB-responsive Tregs may still restrain vascular inflammation, whereas advanced plaques may contain antigen-experienced clones exposed to persistent stimulation. If antigen-presenting cells remain inflammatory and the plaque continues to produce danger signals, a tolerogenic peptide alone may not be sufficient to restore immune regulation. The same ApoB-specific T-cell profile may therefore carry different implications in early, stable, progressive, or the period after acute coronary syndrome. More definitive evidence will require longitudinal studies that combine serial blood sampling, single-cell transcriptomics, TCR clonal tracking, and plaque imaging. In mouse models, fate-mapping and adoptive-transfer experiments could test whether individual ApoB-specific clones move between tolerogenic and inflammatory states. Connecting these trajectories with plaque progression and recurrent events would help assign each cell state to a specific disease stage and clinical outcome.

## 4 Immune tolerance, vaccination, and therapeutic redirection

Because ApoB-specific CD4^+^ T cells can either promote or restrain atherosclerosis, broad T-cell suppression is not an appropriate therapeutic goal. A more selective strategy is to preserve or induce ApoB-specific tolerance while preventing transition toward inflammatory effector states (45). Such an approach could target a disease-relevant self-antigen while retaining immunity to pathogens and tumors. For a chronic disease, durable immune regulation is also more plausible than transient suppression of inflammation (46).

ApoB peptide vaccination is the most developed preclinical approach (47). In *Apoe*^−/−^ mice, vaccination with MHC-II-restricted ApoB peptides reduced atherosclerosis (48). More specifically, immunization with the MHC-II-restricted peptides P101, P102, or P103 reduced aortic plaque and, for P101, increased IL-10, CCR5, and FoxP3 expression in CD4^+^ T cells of the peritoneal cavity and mediastinal lymph node, indicating a regulatory mechanism (49). Subsequent work showed that a clinically applicable adjuvant could support atheroprotective ApoB vaccination in mice (50). Single-cell profiling of P6 vaccine-expanded ApoB-specific T cells further showed clonal expansion dominated by a Treg transcriptional program, alongside a subset retaining Th1-associated features, underscoring the need to verify phenotype as well as cell number after vaccination (51). The intended effect is tolerogenic rather than pathogen-directed immunity, so antigen dose, route, adjuvant, and antigen-presenting cell context must favor regulation rather than effector priming. Human translation remains unresolved because HLA diversity, epitope selection, durability, and the risk of provoking pathogenic responses must all be addressed (12, 52).

Mucosal administration provides another route to tolerance. Intranasal delivery of an ApoB-100 p210 peptide fused to cholera toxin B reduced aortic lesions by approximately 35% in *Apoe*^−/−^ mice and induced IL-10-producing Tr1 cells (53). These findings show that delivery route can strongly influence whether ApoB recognition becomes regulatory or inflammatory. Recent work using ApoB-reactive T-cell receptor-transgenic mice has identified two atheroprotective pathways with different biological consequences. One was linked to humoral immunity and lower cholesterol levels, whereas the other involved Tr1 cells and IL-10 but was accompanied by weaker features of plaque stability. These findings highlight an important point: lesion size alone is not enough to judge antigen-specific interventions, and plaque composition should be assessed in parallel (54). Future platforms, such as tolerogenic nanoparticles and antigen-loaded dendritic cells, will require direct comparison in terms of antigen specificity, durability, manufacturability, and safety before their relative advantages can be defined.

Immune checkpoints and cytokine circuits are potential therapeutic targets, but their effects are context dependent. Direct mouse data show that PD-1/PD-L signaling restrains proatherogenic immunity (42). Other checkpoint pathways, including CTLA-4, and regulatory cytokines such as IL-10 and TGF-β may also support tolerance, but evidence for their selective control of ApoB-specific cells remains limited (43). Systemic manipulation may nevertheless aggravate vascular inflammation or impair host defense, making antigen-restricted delivery preferable whenever feasible. A 2026 proof-of-concept study showed that regulatory T cells engineered with a chimeric antigen receptor against oxidized LDL reduced macrophage foam-cell formation *in vitro* and limited atherosclerotic plaque development in mice (55). This approach targets an oxidized-lipoprotein epitope rather than an ApoB peptide–MHC complex, but it illustrates how antigen-restricted cellular therapy might complement ApoB-specific tolerance strategies.

Maintaining Treg stability remains a major challenge. ApoB-specific regulatory cells may lose FoxP3-associated programs or acquire effector-like features in inflammatory environments, and hyperlipidemia, oxidative stress, and activated antigen-presenting cells can further erode tolerance (16, 30, 56). Effective therapy may therefore need to do more than induce antigen-specific regulation; it may also need to create a tissue environment in which that regulation can persist. Combining tolerance-based therapy with intensive lipid lowering is biologically plausible, because reducing the burden of ApoB-containing lipoproteins should limit ongoing antigen entry and inflammatory stress. However, this combination has not yet been tested directly. It is conceptually distinct from systemic anti-inflammatory treatment such as canakinumab, which reduces inflammatory risk without directly targeting ApoB-specific immunity (1).

Several practical obstacles remain. Because HLA diversity limits the coverage of any single ApoB peptide, epitope selection must balance HLA binding, immunodominance, safety, and the risk of inducing pathogenic rather than regulatory responses (12, 57). Patient selection will also be important. Individuals with high residual inflammatory risk, recurrent events, or measurable inflammatory ApoB-specific T-cell responses despite lipid-lowering therapy may be more suitable for early trials than broadly defined high-risk populations (2, 30).

## 5 Biomarker and translational perspectives

The field now needs biomarkers that capture antigen-specific immunity, not only systemic inflammation. C-reactive protein, lipid measurements, and vascular imaging remain clinically useful, but they cannot determine whether a patient has regulatory or inflammatory ApoB-specific CD4^+^ T-cell responses (58). Peptide–MHC-II tetramers, activation-induced marker assays, single-cell RNA sequencing, paired TCR reconstruction, cytokine profiling, and ApoB-reactive CDR3 motifs can help interrogate this layer of biology (36, 59). Before these tools can be used clinically, they will need standardization, broader HLA coverage, sensitivity testing, and longitudinal validation.

A clinically useful assay should answer several practical questions. Are ApoB-specific CD4^+^ T cells detectable? Do they show regulatory, inflammatory, memory-like, follicular-helper-like, or dysfunctional features? Do their frequency and state correlate with plaque inflammation, recurrent events, or treatment response? Addressing these questions will require longitudinal cohorts that integrate serial blood sampling, plaque imaging, lipid phenotyping, immune profiling, and adjudicated clinical outcomes.

Human studies must also account for HLA diversity. Mouse models allow defined MHC-II-restricted peptides to be tracked, whereas human populations present multiple ApoB epitopes through diverse HLA alleles (12). A translational platform may therefore require panels of tetramers or stimulating peptides that cover common HLA backgrounds. Complementary TCR-based approaches, including conserved ApoB-reactive CDR3 motifs, may help identify convergent antigen-specific responses without requiring prior knowledge of every peptide–HLA pair (36, 60).

Future studies should be designed around mechanism rather than risk status alone. Before ApoB-specific tolerance therapy moves into efficacy trials, early studies should demonstrate target engagement, such as induction of antigen-specific regulatory programs, reduction of inflammatory ApoB-reactive states, or stabilization of these states during lipid-lowering treatment. Observational cohorts should clarify which T-cell states predict plaque burden or recurrent events. Early trials, in turn, should preferentially enroll patients with measurable antigen-specific immune activity rather than unselected high-risk populations. Plaque imaging and immune readouts may provide early pharmacodynamic signals, whereas effects on clinical events will require larger and longer studies (61, 62).

## 6 Conclusion

ApoB-specific CD4^+^ T cells provide a mechanistic link between lipoprotein retention and adaptive immunity in atherosclerosis. Their effects are shaped by MHC-II presentation, chronic self-antigen exposure, plaque inflammation, HLA background, and T-cell plasticity. As a result, the same antigen-specific response may be protective in one disease context and pathogenic in another. The therapeutic goal is therefore not broad immunosuppression, but durable antigen-specific tolerance, ideally combined when appropriate with intensive lowering of ApoB-containing lipoproteins. Progress will depend on standardized tetramer, activation-induced marker, and TCR-based assays, as well as prospective cohorts linking ApoB-specific immune states with plaque biology and clinical events. If these relationships can be established, antigen-specific immunity may become a useful additional dimension in cardiovascular risk stratification and prevention.
